# Supplementary material for: Television watching and cognitive outcomes in adults and older adults: A systematic review and dose-response meta-analysis of observational studies
Source: PLoS One. 2025 Sep 12;20(9):e0323863. doi: 10.1371/journal.pone.0323863 (PMC12431243; doi:10.1371/journal.pone.0323863)
Supplement: S2 Fig — (A) Traffic Light Plot for risk of bias domains. (B) Weighted bar plots of the distribution of risk-of-bias for each domain. (DOCX) [file pone.0323863.s002.docx]

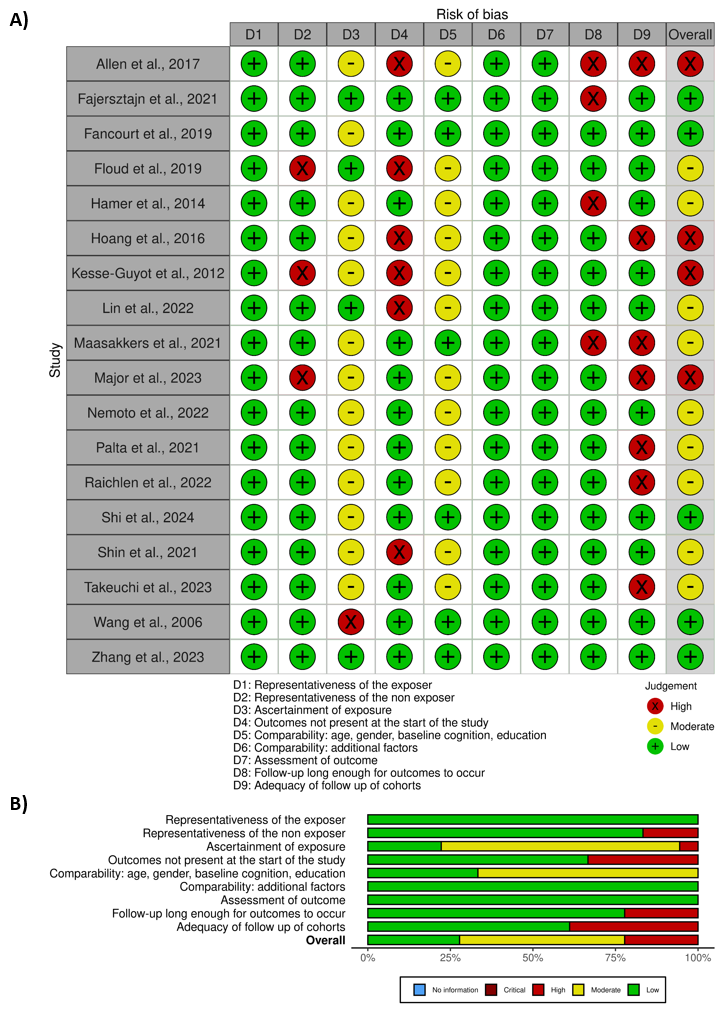


**S2 Fig.** **Risk-of-bias for cohort studies.** (A) Traffic Light Plot for risk of bias domains. (B) Weighted bar plots of the distribution of risk-of-bias for each domain.
