# Supplementary material for: Television watching and cognitive outcomes in adults and older adults: A systematic review and dose-response meta-analysis of observational studies
Source: PLoS One. 2025 Sep 12;20(9):e0323863. doi: 10.1371/journal.pone.0323863 (PMC12431243; doi:10.1371/journal.pone.0323863)
Supplement: S4 Fig — Each circle depicts the logrr and inver_se of cognitive impairment risk at each dose of TV watching time reported in each study. (DOCX) [file pone.0323863.s004.docx]

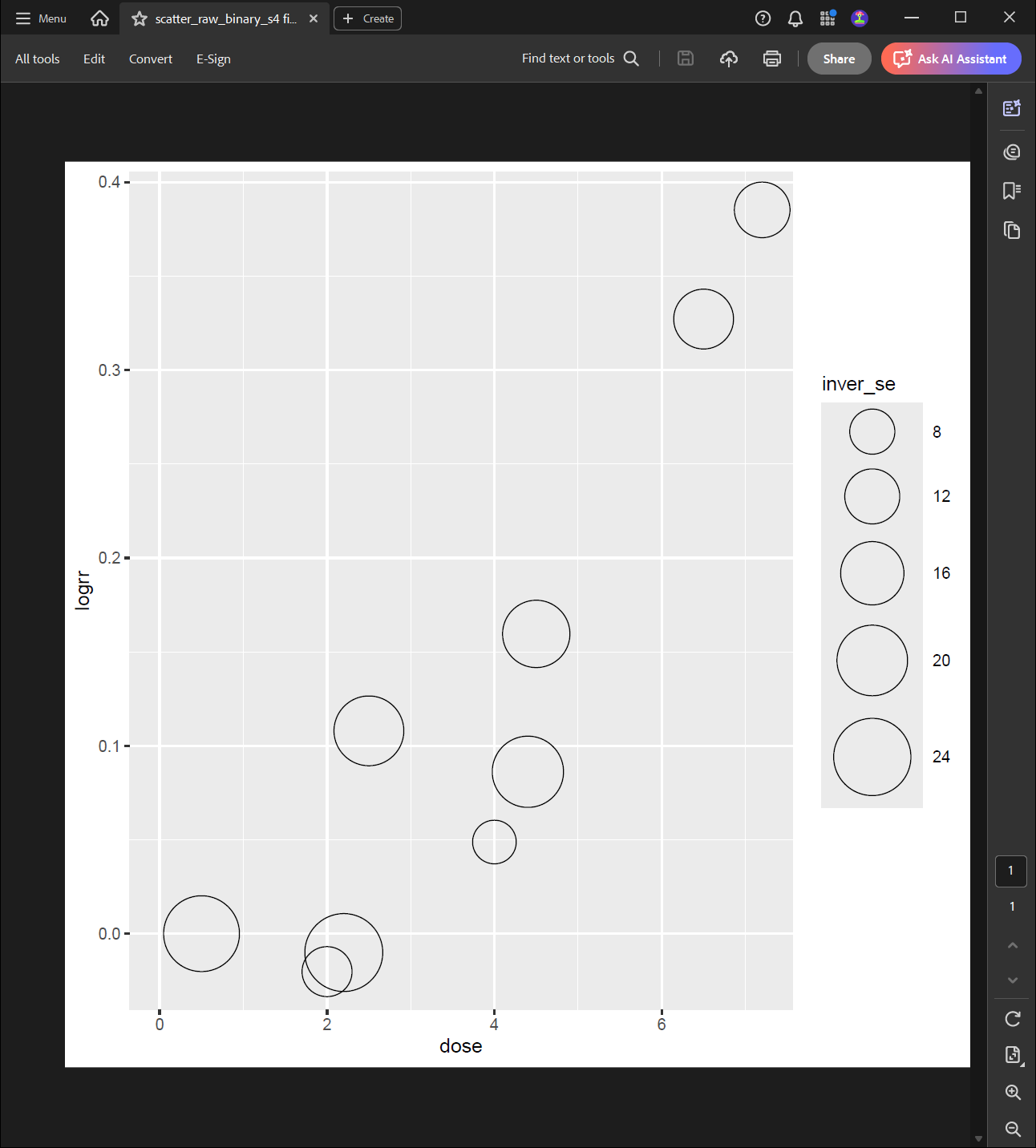


## **S4 Fig.** **Scatter plot of TV watching time (dose; x) and cognitive impairment risk (logrr; y) (4 studies).** Each circle depicts the logrr and inver_se of cognitive impairment risk at each dose of TV watching time reported in each study.
