# Supplementary material for: Television watching and cognitive outcomes in adults and older adults: A systematic review and dose-response meta-analysis of observational studies
Source: PLoS One. 2025 Sep 12;20(9):e0323863. doi: 10.1371/journal.pone.0323863 (PMC12431243; doi:10.1371/journal.pone.0323863)
Supplement: S5 Fig — Conventional meta-analysis of higher versus lower TV watching time and the associated risk of cognitive impairment (11 studies), with subgroup analyses by outcome (upper panel) and study design (lower panel). (DOCX) [file pone.0323863.s005.docx]

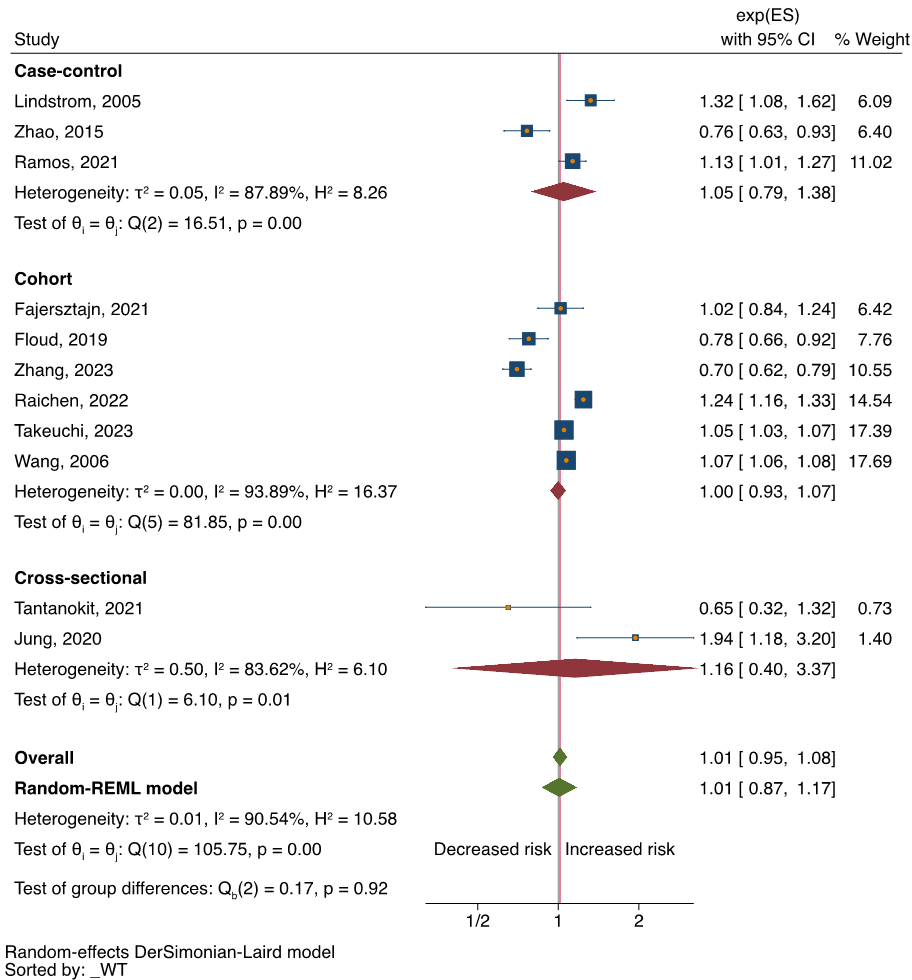


**S5 Fig. Subgroup Meta-Analysis of TV Watching Time and Cognitive Impairment Risk by Outcome and Study Design.** Conventional meta-analysis of higher versus lower TV watching time and the associated risk of cognitive impairment (11 studies), with subgroup analyses by outcome (upper panel) and study design (lower panel).
