# Supplementary material for: Television watching and cognitive outcomes in adults and older adults: A systematic review and dose-response meta-analysis of observational studies
Source: PLoS One. 2025 Sep 12;20(9):e0323863. doi: 10.1371/journal.pone.0323863 (PMC12431243; doi:10.1371/journal.pone.0323863)
Supplement: S6 Fig — Each circle depicts the beta coefficient and inver_se of the change in cognitive score at each dose of TV watching time reported in each study. (DOCX) [file pone.0323863.s006.docx]

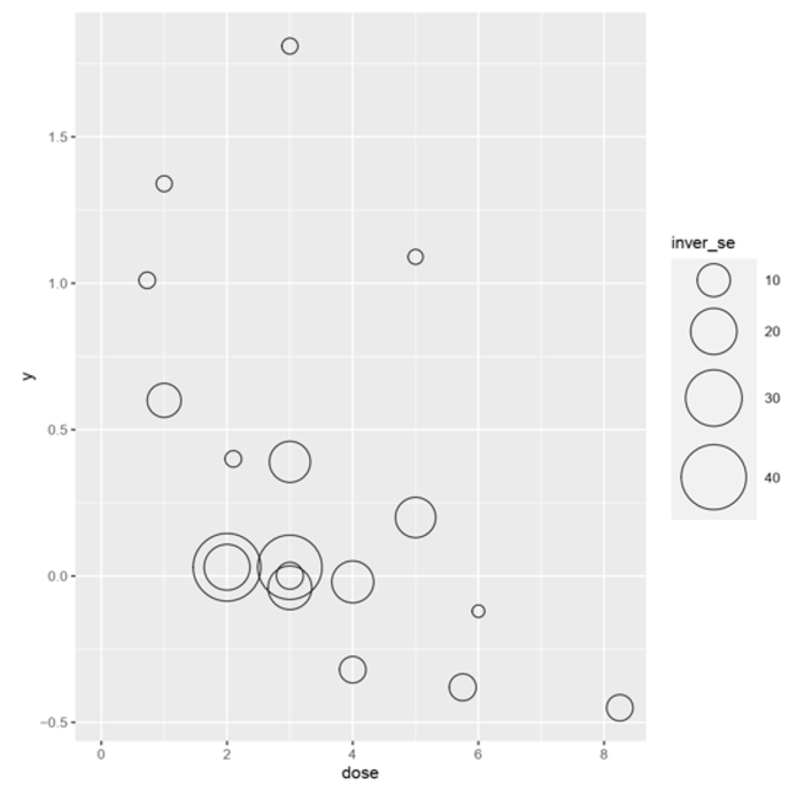


## **S6 Fig**. **Scatter Plot of TV Watching Time (dose; x) and the Change in Cognitive Score (beta coefficient; y).** Each circle depicts the beta coefficient and inver_se of the change in cognitive score at each dose of TV watching time reported in each study.
