# Supplementary material for: Television watching and cognitive outcomes in adults and older adults: A systematic review and dose-response meta-analysis of observational studies
Source: PLoS One. 2025 Sep 12;20(9):e0323863. doi: 10.1371/journal.pone.0323863 (PMC12431243; doi:10.1371/journal.pone.0323863)
Supplement: S7 Fig — (A) Contour-enhanced funnel plot and (B) conventional funnel plot assessing publication bias in the association between TV watching time and risk of cognitive impairment (11 studies). (DOCX) [file pone.0323863.s007.docx]

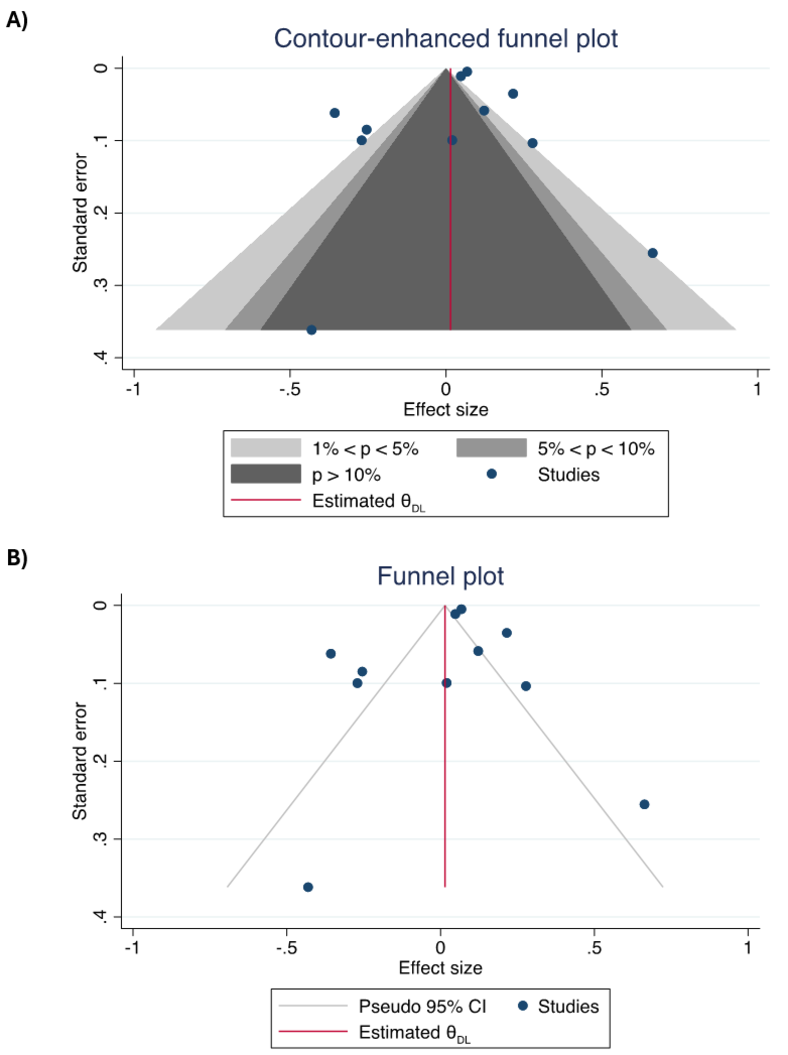


## **S7 Fig. Funnel Plot Analyses for Publication Bias in the Association Between TV Watching Time and Cognitive Impairment Risk**. (A) Contour-enhanced funnel plot and (B) conventional funnel plot assessing publication bias in the association between TV watching time and risk of cognitive impairment (11 studies).
