# Supplementary material for: Television watching and cognitive outcomes in adults and older adults: A systematic review and dose-response meta-analysis of observational studies
Source: PLoS One. 2025 Sep 12;20(9):e0323863. doi: 10.1371/journal.pone.0323863 (PMC12431243; doi:10.1371/journal.pone.0323863)
Supplement: S9 Fig — Leave-one-out analysis evaluating the influence of each individual study on the pooled estimate of the association between TV watching time and (A) risk of cognitive impairment (11 studies) and (B) cognitive score (6 studies). (DOCX) [file pone.0323863.s009.docx]

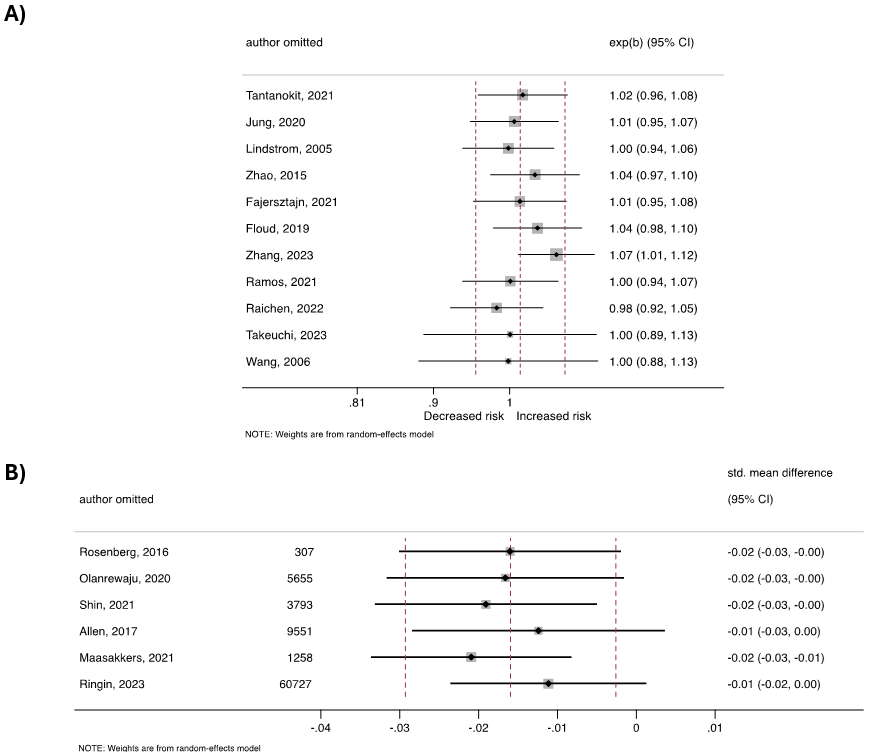


## **S9 Fig.** **Leave-One-Out Sensitivity Analyses for the Association Between TV Watching Time and Cognitive Outcomes.** Leave-one-out analysis evaluating the influence of each individual study on the pooled estimate of the association between TV watching time and (A) risk of cognitive impairment (11 studies) and (B) cognitive score (6 studies).
