# Supplementary material for: Television watching and cognitive outcomes in adults and older adults: A systematic review and dose-response meta-analysis of observational studies
Source: PLoS One. 2025 Sep 12;20(9):e0323863. doi: 10.1371/journal.pone.0323863 (PMC12431243; doi:10.1371/journal.pone.0323863)
Supplement: S2 Table — (DOCX) [file pone.0323863.s011.docx]

**S2 Table.** **Information criteria of each dose-response meta-analysis model.**

| **Model** | **AIC** | **BIC** |
| --- | --- | --- |
| Cognitive impairment risk (binary variable, n=4) | | |
| Linear model | -5.7172 | -8.3309 |
| Quadratic model | -4.1150 | -4.3855 |
| Restricted cubic spline model |  |  |
| 3 Knots* | -7.8001 | -8.0705 |
| 4 Knots* | 1.0364 | -0.8377 |
| 5 Knots* | 7.2085 | 1.7406 |
| Cognitive score (continuous variable, n=7) | | |
| Linear model | -20.1677 | -20.5842 |
| Quadratic model | -28.4294 | -24.5664 |
| Restricted cubic spline model |  |  |
| 3 Knots* | -32.8123 | -28.9493 |
| 4 Knots* | -26.6543 | -20.2819 |
| 5 Knots* | -17.2858 | -8.3390 |

Abbreviation: AIC; Akaike information criterion, BIC; Bayesian information criterion.

**Note:** *Wald test for non-linearity p-values were as follows. For cognitive impairment risk: 0.039 (3 knots), 0.052 (4 knots), and 0.12 (5 knots). For cognitive score 0.33 (3 knots), 0.45 (4 knots), and 0.51 (5 knots).
