# Supplementary material for: Television watching and cognitive outcomes in adults and older adults: A systematic review and dose-response meta-analysis of observational studies
Source: PLoS One. 2025 Sep 12;20(9):e0323863. doi: 10.1371/journal.pone.0323863 (PMC12431243; doi:10.1371/journal.pone.0323863)
Supplement: S3 Table — (DOCX) [file pone.0323863.s012.docx]

**S3 Table.** **Predicted relative risk of cognitive impairment based on dose-response meta-analysis model (n=4).**

| Average TV watching time (hours per day) | Predicted relative risk of cognitive impairment (95% CI) based on 3-knots RCS |
| --- | --- |
| 0 hour per day | 1.00 (Reference) |
| 0.5 hour per day | 1.00 (0.98, 1.02) |
| 2.0 hours per day | 1.01 (0.93, 1.09) |
| 2.5 hour per day | 1.02 (0.94, 1.11) |
| 4.0 hours per day | **1.10 (1.01, 1.20)** |
| 4.5 hour per day | **1.14 (1.05, 1.23)** |
| 6.0 hours per day | **1.30 (1.20, 1.41)** |
| 7.2 hours per day | **1.40 (1.28, 1.54)** |
