# Supplementary material for: Television watching and cognitive outcomes in adults and older adults: A systematic review and dose-response meta-analysis of observational studies
Source: PLoS One. 2025 Sep 12;20(9):e0323863. doi: 10.1371/journal.pone.0323863 (PMC12431243; doi:10.1371/journal.pone.0323863)
Supplement: S4 Table — (DOCX) [file pone.0323863.s013.docx]

**S4 Table. Subgroup analysis of TV watching time and risk of cognitive impairment according to reported effect sizes.**

| Subgroup | Effect size (95% CI), I^2^, p-value for heterogeneity (n) | |
| --- | --- | --- |
|  | Hazard ratio | Odds ratio |
| Pooled effect size | **1.07 (1.02, 1.13), 91.5%, p<0.001 (n=4)** | 0.97 (0.79, 1.20), 88.1%, p<0.001 (n=8) |
| Subgroup analysis |  |  |
| By outcome |  |  |
| Alzheimer’s disease | NA | **1.32 (1.08, 1.62), - (n=1)** |
| Dementia | 1.02 (0.87, 1.21), 94.2%, p<0.001 (n=3) | 0.80 (0.63, 1.01), 0%, p=0.54 (n=2) |
| MCI | **1.07 (1.06, 1.08), - (n=1)** | 0.98 (0.75, 1.27), 91.1%, p<0.001 (n=5) |
| By study design |  |  |
| Cross-sectional | NA | 1.18 (0.75, 1.85), 70.5%, p=0.03 (n=3) |
| Cohort | **1.07 (1.02, 1.13), 91.5%, p<0.001 (n=4)** | 0.83 (0.65, 1.06), 81.0%, p=0.005 (n=3) |
| Case-control | NA | 1.00 (0.59, 1.72), 93.1%, p<0.001 (n=2) |

**Note**: Embolden figures represent statistically significant values (p-value < 0.05). **Abbreviations**: MCI; mild cognitive impairment, NA; not available (due to no information)
