# Supplementary material for: Television watching and cognitive outcomes in adults and older adults: A systematic review and dose-response meta-analysis of observational studies
Source: PLoS One. 2025 Sep 12;20(9):e0323863. doi: 10.1371/journal.pone.0323863 (PMC12431243; doi:10.1371/journal.pone.0323863)
Supplement: S5 Table — (DOCX) [file pone.0323863.s014.docx]

**S5 Table.** **Predicted cognitive score based on dose-response meta-analysis model (n=7).**

| Average TV watching time (hours per day) | Predicted cognitive score (95% CI)  based on 3 knots RCS |
| --- | --- |
| 1 hour per day | -0.0015 (-0.0095, 0.0066) |
| 2 hours per day | -0.0082 (-0.0468, 0.0304) |
| 3 hours per day | -0.0192 (-0.0807, 0.0424) |
| 4 hours per day | -0.0370 (-0.1102, 0.0362) |
| 5 hours per day | -0.0600 (-0.1382, 0.0182) |
| 6 hours per day | **-0.0854 (-0.1682, -0.0026)** |
| 7 hours per day | **-0.1112 (-0.2014, -0.0210)** |
| 8.25 hours per day | **-0.1434 (-0.2463, -0.0405)** |
| I^2^ (%) | 71.8% |
| P-value for heterogeneity | 0.002 |

**Abbreviation:** CI; confidence interval, RCS; restricted cubic spline
