# Supplementary material for: Television watching and cognitive outcomes in adults and older adults: A systematic review and dose-response meta-analysis of observational studies
Source: PLoS One. 2025 Sep 12;20(9):e0323863. doi: 10.1371/journal.pone.0323863 (PMC12431243; doi:10.1371/journal.pone.0323863)
Supplement: S6 Table — (DOCX) [file pone.0323863.s015.docx]

**S6 Table.** **Sensitivity analysis of average TV watching time and predicted cognitive score.**

| Average TV watching time (hours per day) | Predicted cognitive score (95% CI) | |
| --- | --- | --- |
|  | Low-to-moderate RoB (n=5) | Cohort studies (n=4) |
| 1 hour per day | -0.0191 (-0.0579, 0.0197) | 0.0223 (-0.0066, 0.0511) |
| 2 hours per day | -0.0385 (-0.1144, 0.0374) | 0 |
| 3 hours per day | -0.0597 (-0.1633, 0.0440) | -0.0221 (-0.0462, 0.0020) |
| 4 hours per day | -0.0838 (-0.2001, 0.0326) | **-0.0437 (-0.0829, -0.0045)** |
| 5 hours per day | -0.1101 (-0.2293, 0.0091) | **-0.0650 (-0.1157, -0.0143)** |
| 5.75 hours per day | **-0.1307 (-0.2502, -0.0111)** | **-0.0861 (-0.1504, -0.0217)** |
| 7 hours per day | **-0.1651 (-0.2882, -0.0420)** | **-0.1070 (-0.1889, -0.0251)** |
| 8.25 hours per day | **-0.2000 (-0.3309, -0.0682)** | **-0.1332 (-0.2401, -0.0263)** |
| I^2^ (%) | 73.8% | 65.3% |
| P-value heterogeneity | 0.004 | 0.03 |

**Note**: All models were fitted using restricted cubic spline with 3 knots. **Abbreviation**: CI; confidence interval, RoB; risk of bias
