# Supplementary material for: Television watching and cognitive outcomes in adults and older adults: A systematic review and dose-response meta-analysis of observational studies
Source: PLoS One. 2025 Sep 12;20(9):e0323863. doi: 10.1371/journal.pone.0323863 (PMC12431243; doi:10.1371/journal.pone.0323863)
Supplement: S7 Table — (DOCX) [file pone.0323863.s016.docx]

**S7 Table.** **Certainty of findings according to GRADE.**

| Outcomes | Impact | No. of participants (studies) | Certainty of the evidence (GRADE) |  |
| --- | --- | --- | --- | --- |
|  |  |  |  |  |
| Cognitive impairment (DRMA) | The risk of cognitive impairment increases nonlinearly with longer TV-watching time, particularly beyond 4 hours per day. | (4 non-randomized studies) | ⨁⨁⨁◯ Moderate^a,b^ |  |
| Cognitive score (DRMA) | Watching TV for six hours or more per day is associated with a significantly decreased cognitive score. | (7 non-randomized studies) | ⨁⨁◯◯ Low^a,b,c^ |  |
| Cognitive impairment (SRMA) | Higher TV watching, compared to lower TV watching, is not associated with the risk of cognitive impairment. The pooled relative risk is 1.01 (95% CI: 0.95, 1.08). | (11 non-randomized studies) | ⨁◯◯◯ Very low^c,d,e,f^ |  |
| Cognitive score (SRMA) | Higher TV watching, compared to lower TV watching, is associated with a slight decrease in cognitive score: the standardized beta coefficient was -0.02 (95% CI: -0.03, -0.003). | (6 non-randomized studies) | ⨁◯◯◯ Very low^b,d,f,g^ |  |
| **Abbreviation:** DRMA; dose-response meta-analysis, SRMA; systematic review meta-analysis, CI; confidence interval  **GRADE Working Group grades of evidence** **High certainty:** we are very confident that the true effect lies close to that of the estimate of the effect. **Moderate certainty:** we are moderately confident in the effect estimate: the true effect is likely to be close to the estimate of the effect, but there is a possibility that it is substantially different. **Low certainty:** our confidence in the effect estimate is limited: the true effect may be substantially different from the estimate of the effect. **Very low certainty:** we have very little confidence in the effect estimate: the true effect is likely to be substantially different from the estimate of effect. | | | |  |

Explanations

a. Although some of the included studies have a high risk of bias, excluding it does not change the results.

b. Less than 10 studies were included in the analysis, which could lead to imprecise findings due to the small number of studies.

c. There is a high degree of statistical heterogeneity with unknown sources.

d. Results from sensitivity and subgroup analysis are not consistent with the main findings.

e. The main findings are null, with the corresponding confidence interval covering both directions of the association.

f. There are some studies that can influence the main findings.

g. There is a high degree of statistical heterogeneity, but the source of the heterogeneity has been identified.
