# Supplementary material for: Television watching and cognitive outcomes in adults and older adults: A systematic review and dose-response meta-analysis of observational studies
Source: PLoS One. 2025 Sep 12;20(9):e0323863. doi: 10.1371/journal.pone.0323863 (PMC12431243; doi:10.1371/journal.pone.0323863)
Supplement: S1 File — (DOCX) [file pone.0323863.s017.docx]

**S1 File. Supplementary methods**

**Television watching and cognitive outcomes in adults and older adults: A systematic review and dose–response meta-analysis of observational studies**

Hattapark Dejakaisaya^1^, Wiriya Mahikul^1^, Nat Na-ek^2-4^, Chanawee Hirunpattarasilp^1,5*^

^1^Princess Srisavangavadhana Faculty of Medicine, Chulabhorn Royal Academy, Bangkok, Thailand

^2^Division of Pharmacy Practice, Department of Pharmaceutical Care, School of Pharmaceutical Sciences, University of Phayao, Phayao, Thailand

^3^Pharmacoepidemiology, Social and Administrative Pharmacy (P- SAP) Research Unit, School of Pharmaceutical Sciences, University of Phayao, Phayao, Thailand

^4^Unit of Excellence on Cardiovascular Archive Research and Clinical Epidemiology, School of Pharmaceutical Sciences, University of Phayao, Phayao, Thailand

^5^Department of Neurology, University Hospitals Cleveland Medical Center, Cleveland, Ohio, United States of America

* Corresponding author

E-mail: chanawee.hir@cra.ac.th (CH)

**Table of contents**

[**Search strategy** 3](#_Toc201764164)

[**Cochrane** 3](#_Toc201764165)

[**PsycINFO** 3](#_Toc201764166)

[**Scopus** 3](#_Toc201764167)

[**Ovid for MEDLINE** 3](#_Toc201764168)

[**EMBASE** 3](#_Toc201764169)

[**Web of Science** 3](#_Toc201764170)

[**Materials and methods** 4](#_Toc201764171)

[**Assessment of Bias of Individual Study** 4](#_Toc201764172)

[**Statistical Analysis** 4](#_Toc201764173)

[**Data Items** 4](#_Toc201764174)

[**Reference** 4](#_Toc201764175)

# **Search strategy**

## **Cochrane**

((Television) OR (TV)) AND ((cognitive function) OR (neuropsychological test) OR (dementia)) AND ((adult) OR (elderly))

## **PsycINFO**

(television OR TV) AND (cognitive function OR cognition OR MCI OR cognitive impairment OR intellect OR attention OR memory OR executive function OR neuropsychological test OR dementia OR Addenbrooke OR alzheimer OR MMSE OR Mini Mental State Examination OR Montreal cognitive assessment) AND (adult OR aged OR elderly)

## **Scopus**

TITLE-ABS-KEY (Televi* OR TV) AND

TITLE-ABS-KEY (cognit* OR MCI OR intellect* OR attention OR memory OR executive function OR neuropsychologic* OR dement* OR Addenbrooke OR Alzheimer OR MMSE OR Mini Mental State Examination OR Montreal cognitive assessment) AND

TITLE-ABS-KEY (Adult*OR Aged* OR elderl*)

## **Ovid for MEDLINE**

(exp television viewing/ or exp television/) and (exp cognition/ or exp cognition assessment/ or exp cognitive defect/ or exp dementia/ or dementia assessment/ or exp cognitive dysfunction/ or exp Alzheimer/) and (exp human/) and (exp aged/ or exp adult/ or elderl*)

## **EMBASE**

('television viewing'/exp OR 'television'/exp) AND ('cognition'/exp OR 'cognition assessment'/exp OR 'cognitive defect'/exp OR 'dementia'/exp OR 'dementia assessment'/exp OR 'cognitive defect'/exp OR 'Alzheimer disease'/exp) AND ('human'/exp) AND ('aged'/exp OR 'adult'/exp OR elderl*)

## **Web of Science**

TS = (Televi* OR TV) AND (cognit* OR MCI OR intellect* OR attention OR memory OR executive function OR neuropsychologic* OR dement* OR Addenbrooke OR Alzheimer OR MMSE OR Mini Mental State Examination OR Montreal cognitive assessment) AND (Adult* OR Aged* OR Elderl*)

# **Materials and methods**

## **Assessment of Bias of Individual Study**

Included studies were assessed for bias using NOS by two independent reviewers (HD, WM). Any disagreements were resolved through discussion. The studies were then classified as high (0-5 NOS points), moderate (6-7 NOS points), or low (8-9 NOS points) risk of bias. Risk-of-bias Visualization (robvis) software was used to outline the risk of bias appraisal[1].

## **Statistical Analysis**

### **Data Items**

Information from each study was extracted on the following aspects: 1) demographics of the included study (i.e., authors, years of publication, country of setting, article type, and study design); 2) study population (i.e., sample size, percentage of males, mean age at baseline, mean follow-up time, inclusion and exclusion criteria); 3) exposure (i.e., quantification of TV watching and unit of measurement); and 4) outcome (type of outcome, testing criteria, adjusted and unadjusted effect size, corresponding confidence interval, standard error, and p-value).

# **Reference**

1. McGuinness LA, Higgins JPT. Risk-of-bias VISualization (robvis): An R package and Shiny web app for visualizing risk-of-bias assessments. Res Synth Methods. 2021;12(1):55-61.
