# Supplementary material for: Television watching and cognitive outcomes in adults and older adults: A systematic review and dose-response meta-analysis of observational studies
Source: PLoS One. 2025 Sep 12;20(9):e0323863. doi: 10.1371/journal.pone.0323863 (PMC12431243; doi:10.1371/journal.pone.0323863)
Supplement: Attachment — Submitted filename: Response to Reviewers.docx [file pone.0323863.s018.docx]

**Response to Reviewers**

PONE-D-25-04250

Television watching and cognitive outcomes in adults and older adults: A systematic review and dose–response meta-analysis of observational studies

PLOS ONE

**1. Reviewer #1: The paper is interesting. However, there are some concerns from a statistical perspective. Certainty of findings grade in supplement were all very low to moderate concerning evidence of certainty which may indicate that the association sought by the investigators is not very strong.

1.1 All the included studies were observational in design, meaning that the authors had doubt of the associations examined as they note, “particularly the influence of residual confounders and reverse causality (e.g., individuals in the subclinical stage of cognitive impairment may spend most of their time watching TV due to physical limitations)—cannot be ruled out. Therefore, causality cannot be inferred from our findings.” This use of the term , causality, by the authors is inappropriate as the effect size measures in this study and most statistical studies involves evidence of association and not causality. Thus causality is not in play, especially in a meta-analysis, which is primarily exploratory or suggestive. The authors note correctly themselves that regarding the association between TV-watching time and cognitive score, they found a nonlinear relationship via a three-knot restricted cubic spline model, which was the best-fitting model in the dose–response meta-analysis according to the AIC and BIC in Table 3 of the supplement. Likewise, they give the same quantitative interpretation correctly for the Analysis of the risk of cognitive impairment via the term association. That is as far as they can go in the interpretation. They cannot assess causality with their approach. They should rethink this interpretation and stay with association, strong or weak.**

We thank the reviewer for the comment. We acknowledge the limitations of observational studies and agree that our findings can only indicate associations, not causality. In response to the reviewer’s concern, we have revised the manuscript to remove or rephrase any mention of "causality" to ensure that the interpretation of our results remains within the appropriate scope of observational research. Specifically, we now consistently use the term “association” throughout the manuscript to reflect the nature of the evidence.

**1.2 In general the synthesis of the findings and its organization was done and interpreted correctly including the risk of bias assessment.**

We thank the reviewer for the positive comment.

**1.3. The analysis requires much clarification. The authors note in the analysis portion that, where possible, fully adjusted effect sizes were used. This is not clear . What adjustment? There is reference to confounders and causes for a weak or strong association in some cases. However, there is no real detailed evidence of relevant confounder discussion (clinical or demographic in addition to the self report limitations noted by the authors) in the text or supplement.**

To improve clarity, we have now explained the logic behind using the fully adjusted effect sizes (page 8, para 2) and listed the covariates adjusted for in each included study in Table 1:
“Where possible, we used the effect sizes from models that included the most comprehensive set of covariates reported in each primary study to account for potential cofounders such as age, sex, education, socioeconomic status, lifestyle factors (e.g. physical activity, smoking), and other comorbidities. These factors, such as older age, female sex and lower education attainment, can negatively affect the cognitive outcomes [40]. Details of covariate adjusted for in each study are provided in Table 1.”

**1.4. Also, the I squares are given with p-values in the Figures 2 and 3 or in the supplement with no explanation of the causes of significance , if any. The funnel plots are well done and presented in the supplement. However, the asymmetry (Figure 7A) should be explained. Also, the authors should explain in more detail the difference in the graphical presentations of Supplementary Figures 6 and 7 vs. Supplementary Figures 8 and 9.**

We have now added the following text to discuss the significant heterogeneity, suggested by I² and its p-values, on page 27, para 1:

“Second, some results showed a significant degree of heterogeneity. Consequently, for some findings, the certainty of the evidence was rated as low to very low according to GRADE, such that readers should exercise caution when interpreting the findings. Our subgroup and sensitivity analyses (Table 2) suggest that study design, outcome type, reported effect sizes, and risk of bias were not the primary contributors to the heterogeneity. This persistent heterogeneity across subgroups suggests that other factors, such as unmeasured confounders, may contribute. These may include differences in TV assessment methods (e.g., self-reported hours vs. categorical measures), regional variations in viewing habits, and the use of different cognitive measures and scales (e.g., MMSE vs. MoCA vs self-report). These factors likely contributed to variability beyond study design or risk of bias; thus, the pooled estimates should be interpreted with caution. Interestingly, in the leave-one-out analysis, (Zhang et al., 2023 [66]) appears to be a potential influential source, as its exclusion shifts the pooled estimate, suggesting it contributes to observed heterogeneity.”

Regarding the asymmetry of the funnel plot (Supplementary Figure 7), Egger’s test indicated no statistically significant publication bias (p-value = 0.43). The asymmetry is more likely to be driven by between-study heterogeneity and variation in study precision.

The Egger’s test result is stated on page 23, para 1 as follows:

“Visual inspection of the contour-enhanced and conventional funnel plots for cognitive-impairment risk (S7A & B Fig) suggested some asymmetry; however, Egger’s test indicated no statistically significant publication bias (p-value = 0.43).”

The cause of asymmetry is discussed on page 28, para 1:

“The apparent funnel-plot asymmetry is more plausibly driven by between-study heterogeneity and variation in study precision than by small-study publication bias. In addition, our comprehensive search strategy—covering six major databases (Cochrane, Ovid MEDLINE, Ovid Embase, PsycINFO, Scopus, and Web of Science)—reduces the likelihood that relevant studies were missed, further minimizing the chance that publication bias explains the pattern observed.”

The graphical differences between Supplementary Figures 6-9 reflect the different outcomes assessed. We have now changed the figure legend for Supplementary Figure 6 to further explain the graph as follows:
“S6 Fig. Scatter Plot of TV Watching Time (dose; x) and the Change in Cognitive Score (beta coefficient; y). Each circle depicts the beta coefficient and inver_se of the change in cognitive score at each dose of TV watching time reported in each study.”

Supplementary Figures 7 and 8 showed funnel plots with and without contour enhancements to evaluate potential publication bias for cognitive impairment risk and cognitive scores, respectively. The difference is stated on page 23, para 1 as follows:

“Visual inspection of the contour-enhanced and conventional funnel plots for cognitive-impairment risk (S7A & B Fig) suggested some asymmetry; however, Egger’s test indicated no statistically significant publication bias (p-value = 0.43). Likewise, both the contour-enhanced funnel plot (S8A Fig) and the classical funnel plot (S8B Fig) of the cognitive score outcome also showed no apparent evidence of publication bias, with a p-value of 0.56 derived from Egger’s test.”

Supplementary Figure 9 shows leave-one-out sensitivity analyses for both outcomes, illustrating the influence of individual studies on the overall pooled estimates. The leave-one-out analysis is explained on page 9, para 2:

“Additionally, the influence of each study was examined by performing a leave-one-out analysis.”

And the result of the Supplementary Figure 9 has been discussed on page 23, para 1:

“In the leave-one-out analysis (S9 Fig), although most studies did not influence the findings, three studies might have dominated the main results. Omitting one study (Zhang et al., 2023 [66]) from the meta-analysis of cognitive impairment risk (S9A Fig) changed the results from null to significant (1.07 [95% CI: 1.01, 1.12]). In contrast, leaving out either of two studies (Shin et al., 2021 [63] and Maasakkers et al., 2021 [58]) in the meta-analysis of cognitive score reverted the results to null (S9B Fig).“

**1.5. Also, there should be a caution about few cross sectional studies on the Forest plots (Figure 3B) and care in interpreting the p-values when there are so few studies on a Forest plot. The entire document should be edited to be sure relevant detail is provided to the reader.**

We agree with the reviewer. We have added the following sentence to the limitation section in our discussion (page 28, para 1):
“Fourth, it should be noted that forest plots with a limited number of studies (e.g., subgroup analyses with ≤2 studies) as shown in Fig 3B should be interpreted with caution. P-values may not reliably indicate true between-group differences under these conditions.”

**2. Reviewer #2: The manuscript presents a systematic review and meta-analysis to investigate the association between television watching and cognitive outcomes in adults and older adults. The topic is of significant relevance, the PRISMA checklist is followed, and the supplement is comprehensive. The inclusion of dose–response analyses and a large data set are major strengths. To improve transparency, reproducibility, and reader accessibility, I have several suggestions as detailed below. Overall, this is a valuable and important manuscript that will benefit from greater clarity and integration of its supplementary material.**

**2.1 Methods
2.1.1 Data Extraction: Please summarize the outcome selection hierarchy and its rationale in the main Methods for clarity. Consider moving a summary of the main variables extracted (as per the supplement) to the main text.**

Thank you for your feedback. As per your request, we have moved the following summary of the main variables extracted from the supplementary file to the data extraction section in the main text (page 6, para 2):
“Studies with overlapping populations or from the same database were ranked based on a designed hierarchy and the studies with the highest hierarchical score were included. Briefly, studies were ranked based on 1) the most relevant outcomes (e.g. dementia, cognitive impairment, and cognitive score); 2) sample size (largest); and 3) year of publication (latest), respectively. For articles with multiple levels of television viewing, all data were collected to analyze the dose-response relationship. For studies that report both cognitive score and MCI/dementia risk, we collected both outcomes for their respective meta-analyses.”

**2.1.2 Assessment of Bias: Add a brief explanation of the NOS domains so that readers unfamiliar with the tool can understand your assessment. For transparency, summarize the distribution of risk categories (low, moderate, high risk of bias) in the main text.**

We have added the following explanation of the NOS domains to the methods section (page 6, para 3):
“The Newcastle–Ottawa Quality Assessment Scale (NOS) was applied to evaluate and analyze the methodological quality of each study [28, 29]. Three domains were evaluated: selection, comparability, and outcome/exposure assessment. Two reviewers (HD, WM) independently scored studies as low (8–9 points), moderate (6–7 points), or high (0–5 points) risk of bias. Disagreements were resolved by discussion. A summary of risk levels and visualizations was generated using robvis [30].”

**2.1.3 Statistical Analysis: Clearly summarize the hierarchy and decision process for handling multiple outcomes in the main Methods.**

We have moved the following text from the supplementary to the main text (page 7, para 2 – page 8, para 1):
“To avoid duplication issues, we analyzed only one outcome from each study that reported more than one outcome with the same type of variable (continuous or binary), using the following hierarchy: 1) If both cohort and cross-sectional results were reported [36], we used the cohort results; 2) If each outcome was reported along with a cumulative one [37], we selected the cumulative one; 3) If both short- and long-term outcomes were reported [23], we chose the long-term one; 4) If each outcome was reported separately without a cumulative one [38] [39], we used the outcome with the smallest variance.”

**2.2 Results
2.2.1 Please move a summary table of study characteristics into the main Results for quick reader reference.**

As requested, we have moved the summary table of study characteristics from the supplementary materials into the main results section as Table 1 to facilitate easier access and quick reference for readers.

**2.2.2 Given the high heterogeneity in many analyses, please provide a more granular exploration or discussion of possible sources (e.g., differences in TV assessment methods, region, cognitive test used).**

We have added the following discussion regarding heterogeneity in the discussion section (page 27, para 1):

“Second, some results showed a significant degree of heterogeneity. Consequently, for some findings, the certainty of the evidence was rated as low to very low according to GRADE, such that readers should exercise caution when interpreting the findings. Our subgroup and sensitivity analyses (Table 2) suggest that study design, outcome type, reported effect sizes, and risk of bias were not the primary contributors to the heterogeneity. This persistent heterogeneity across subgroups suggests that other factors, such as unmeasured confounders, may contribute. These may include differences in TV assessment methods (e.g., self-reported hours vs. categorical measures), regional variations in viewing habits, and the use of different cognitive measures and scales (e.g., MMSE vs. MoCA vs self-report). These factors likely contributed to variability beyond study design or risk of bias; thus, the pooled estimates should be interpreted with caution. Interestingly, in the leave-one-out analysis, (Zhang et al., 2023 [66]) appears to be a potential influential source, as its exclusion shifts the pooled estimate, suggesting it contributes to observed heterogeneity.”

**2.2.3 Review, update, and ensure consistency for all figure/table numbers and legends so that all are referenced appropriately in the text.**

We have reviewed and updated all figure and table numbers, legends, and corresponding in-text references to ensure consistency and accuracy throughout the manuscript. All figures and tables are now appropriately cited in the main text, and their legends have been revised for clarity and completeness.

**2.2.4 Clarify and standardize decimal place reporting.**

Thank you for your comment regarding the standardization of decimal places for p-values and effect sizes. We acknowledge PLOS ONE’s guideline that p-values greater than or equal to 0.001 should be reported as exact values, and p-values less than 0.001 may be expressed as p < 0.001.

Accordingly:

- We reported p-values < 0.001 as p < 0.001.
- For p-values between 0.001 and 0.01, we used three decimal places to retain precision.
- For p-values ≥ 0.01, we used two decimal places, which aligns with common practice in medical literature (e.g., JAMA, NEJM) and retains the precision necessary for interpretation without misrepresentation.

This approach ensures consistency and balances interpretability with practical constraints.

For effect sizes such as beta coefficients, we standardized the reporting to two decimal places throughout the manuscript. In instances where rounding to two decimal places yields values like 0.00 or –0.00 (e.g., -0.003), we reported them to three decimal places to avoid misinterpretation.

We hope this explanation is acceptable and appreciate your attention to detail, which has helped enhance the quality of our submission.

**2.2.5 Use color schemes/line types in figures that are clear to all readers.**

We understand the importance of using accessible and reader-friendly visual elements in figures. However, due to limitations in the software version used for the analysis and figure generation, we are unable to modify the color schemes and line types. We would also like to note that the current visual presentation does not affect the interpretation of the effect sizes or statistical results presented. For these reasons, we have retained the original color scheme and line types. We appreciate your understanding on this matter.

**2.2.6 The main text cites references in mixed formats; please standardize to one citation style throughout.**

We have now reviewed the manuscript and standardized all in-text citations and references to ensure consistency with the citation style required by PLOS ONE. The revised manuscript now adheres to a uniform citation format throughout the main text, tables, and figure legends.

**2.2.7 Key dose–response, subgroup, and sensitivity findings should be highlighted within the main Results section, not just in the supplement.**

In our revised manuscript, we have ensured that the key findings for dose-response, subgroup, and sensitivity findings are clearly stated in the manuscript. The result of the dose-response meta-analysis of the risk of cognitive impairment can be found on page 19, para 1. The dose-response result of the cognitive score is stated on page 21, para 1. Subgroup and sensitivity findings for risk of cognitive impairment and cognitive score can be found on page 19, para 2, and page 21, para 2 respectively.

**2.3. Discussion
2.3.1 The discussion focuses solely on "television" as a sedentary behavior. Please also discuss whether findings may generalize to other screen-based activities (e.g., internet videos, streaming platforms) and address the limitations of this extrapolation.**

We thank the reviewer for the suggestion. We have added the following discussion on this topic on page 26, para 2:

“Since this study focuses on the relationship between TV watching (a commonly used marker for sedentary behavior) and cognition, it may be possible to apply the results from this study to similar sedentary activities, such as watching internet videos and using streaming platforms. However, it is important to note the different variables associated with each type of sedentary activity, for example, some activities may be more appealing to certain age and gender groups than others. In addition, some activities, such as watching internet videos, might be associated with a higher degree of interaction with the user than watching television. Factors such as these may alter the relationship between different sedentary behavior and cognition [40]. Thus, the results from this study must be extrapolated with caution.”

**2.3.2 Briefly consider whether the content of TV programming might matter (e.g., educational vs. non-educational, passive vs. active).**

There is currently a lack of evidence on this specific topic. We have added the following sentence to the future directions section (page 28, para 2):

“Additionally, future studies should consider the relationship between different types of TV programming on cognitive decline in adults as there is currently a lack of evidence on this specific topic.”
